# Giant dielectric and magnetoelectric responses in insulating nanogranular films at room temperature

**DOI:** 10.1038/ncomms5417

**Published:** 2014-07-22

**Authors:** Nobukiyo Kobayashi, Hiroshi Masumoto, Saburo Takahashi, Sadamichi Maekawa

**Affiliations:** 1 Research Institute for Electromagnetic Materials, 2-1-1,Yagiyama-minami, Taihaku-ku, Sendai 982-0807, Japan; 2 Frontier Research Institute for Interdisciplinary Sciences, Tohoku University, 6-3, Aramaki aza Aoba, Aoba-ku, Sendai 980-8578, Japan; 3 Institute for Materials Research, Tohoku University, 2-1-1 Katahira, Aoba-ku, Sendai 980-8577, Japan; 4 Advanced Science Research Center, Japan Atomic Energy Agency, 2-4, Shirakata Shirane, Tokai-mura, Ibaraki 319-1195, Japan

## Abstract

The electric and magnetic properties of matter are of great interest for materials science and their use in electronic applications. Large dielectric and magnetoelectric responses of materials at room temperature are a great advantage for electromagnetic device applications. Here we present a study of FeCo-MgF nanogranular films exhibiting giant dielectric and magnetoelectric responses at room temperature; with dielectric constant *ε*′=490 and magnetoelectric response Δ*ε*′/*ε*′_0_=3%. In these films, Fe-Co alloy-based nanometer-sized magnetic granules are dispersed in a Mg-fluoride-based insulator matrix. Insulating nanogranular films are a new class of multifunctional materials. The giant responses are caused by spin-dependent charge oscillation between magnetic granules via quantum-mechanical tunnelling. A possible application of such insulating nanogranular materials with giant response is in the construction of a tunable device, in which impedance components such as capacitance and inductance are tunable at room temperature.

## Abstract (web summary)

The electric and magnetic responses of matter are of interest for their use in electronic applications. Here, the authors find a large dielectric and magnetoelectric response in FeCo-MgF nanogranular films, caused by quantum mechanical tunnelling oscillation between magnetic granules.

## Graphical abstract

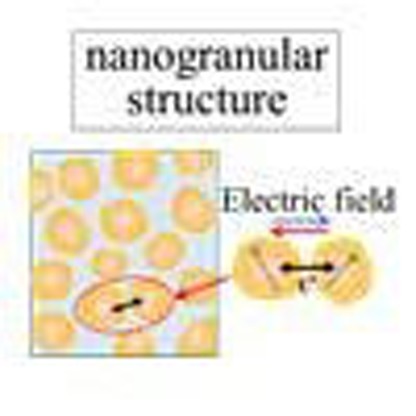

Response of matter to electric and magnetic fields is of great interest in physics^1^,^2^,^3^ and its applications^4^,^5^. A number of candidates have been reported as magnetoelectric systems such as oxides^6^,^7^ and quantum dots^8^,^9^. However, the magnetic field response of these systems is too small at room temperature from the viewpoint of applications. This is because the magnetic transition temperature of reported oxide materials possessing the magnetoelectric effect is much lower than room temperature or the effect operates only at low temperatures.

Insulating nanogranular films consist of magnetic nanometer-sized metal granules and an insulator matrix, and exhibit various functional properties depending on the composition ratio of the metal granules and the insulator matrix in the film^10^,^11^. They have significant practical advantages of easy fabrication and thermal stability^12^ and have been applied in magnetic sensors^13^,^14^.

Magnetoelectric coupling shows great promise for achieving tunable devices^15^,^16^,^17^,^18^,^19^ where the impedance in a specific frequency range can be changed according to the input signal. Recently, such devices need to operate in a wide frequency band that includes both digital and analogue broadcasts. Up to now, such tunable devices have been prepared by combining two or more materials of different properties. However, if a magnetoelectric material with multifunctional properties can be utilized, the device structures may become simplified and an expansion in further applications of the devices can be expected.

In this work, we have studied the dielectric properties of insulating nanogranular materials, reporting specifically on the magnetoelectric effects in FeCo-MgF nanogranular films. Fe-Co alloy has the largest magnetization, and good interface between Fe-Co nanogranules and MgF_2_ matrix is obtained^14^. These properties result in the giant dielectric and magnetoelectric response in FeCo-MgF nanogranular films at room temperature. The experimental results are well explained by spin-dependent charge oscillation caused by quantum mechanical tunnelling between magnetic granules.

## Results

### Structure of the FeCo-MgF nanogranular films

**Figure 1:**
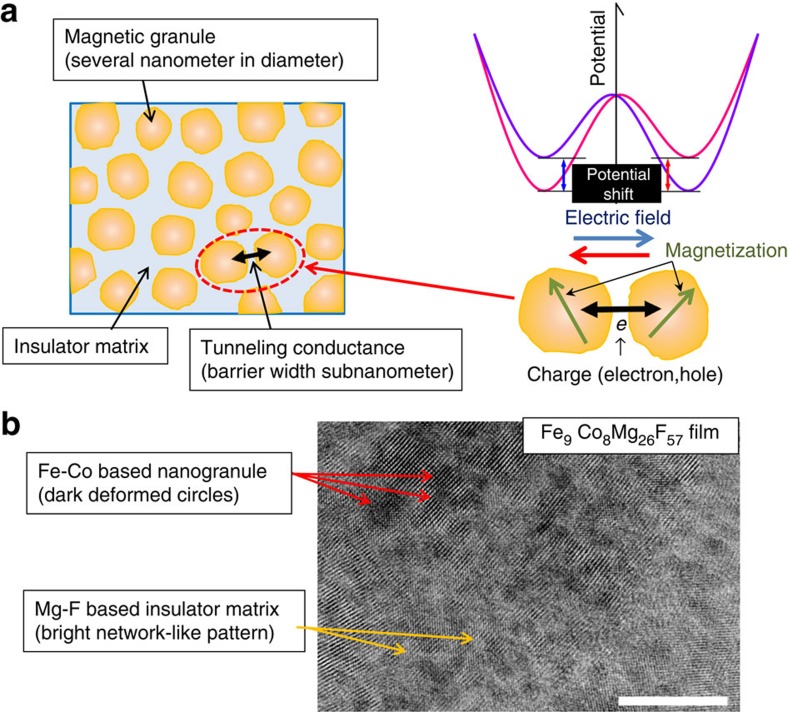
Structure and electric polarization of a nanogranular film. (**a**) A schematic of a nanogranular film with nanometer-sized granules dispersed in an insulator matrix. The right-side figure in **a** represents a schematic of the electric potential profile of two granules in an ac electric field that generate oscillating transition of an electric charge carrier (electron, hole) between them via quantum-mechanical tunnelling through the thin insulating barrier. The oscillating transition of the charge-carrier with spin depends on the relative orientation of the magnetization of the granules. This spin-dependent charge oscillation is the origin of the dielectric and magnetoelectric (magneto-dielectric) responses of the nanogranular films. (**b**) A high-resolution transmission electron microscope image (in plain) of Fe_9_Co_8_Mg_26_F_57_ (Fe+Co=17 at.%) film of a typical sample (scale bar, 10 nm).

Figure 1a illustrates a nanogranular structure, which consists of nanometer-sized magnetic granules dispersed in an insulator matrix. Figure 1b shows a high-resolution transmission electron microscope image obtained from Fe_9_Co_8_Mg_26_F_57_ (Fe+Co=17 at.%) thin film, which is one of the samples produced in this study. In this micrograph, many small dark and deformed circles with diameters ranging from about 2 to 4 nm are observed. In addition, a bright network-like pattern covering the whole area is identified. The dark deformed circles are Fe-Co alloy-based granules, and the small bright section indicates the Mg-fluoride-based matrix.

### Dielectric properties and magneto-dielectric effects

**Figure 2:**
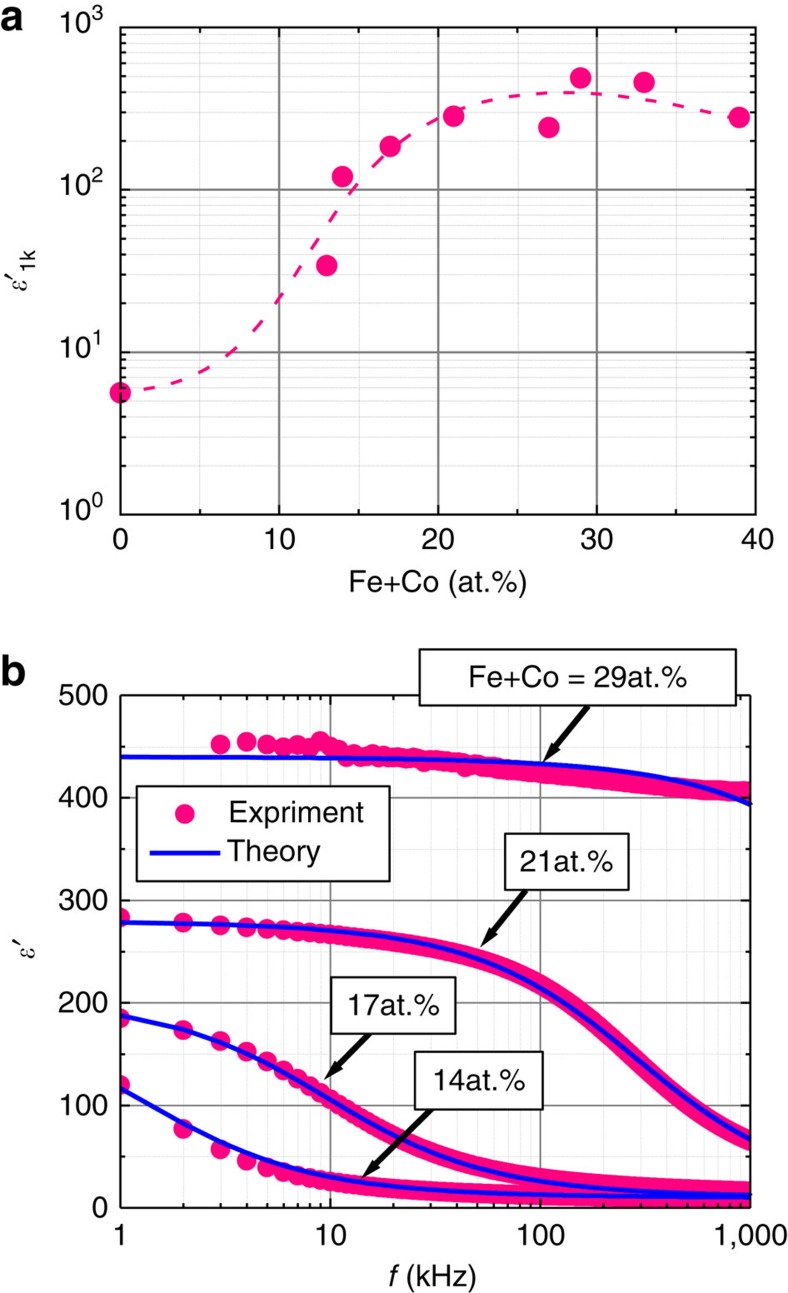
Dielectric properties of the FeCo-MgF nanogranular films. (**a**) Dielectric constant at 1 kHz (ε′_1k_) measured using an LCR meter vs the composition ratio of Fe+Co in FeCo-MgF films. The value of ε′_1k_ is 5.6 at 0 at.%, and increases with increasing Fe+Co content, attaining 490 at 30 at.%. (**b**) Dielectric constant ε′ vs frequency *f* of FeCo-MgF films for Fe+Co=14, 17, 21, and 29 at.% measured in the frequency range of 1 kHz–1 MHz. The red dots represent the experimental results, and the blue solid lines represent the theoretical results obtained from calculations based on the spin-dependent dielectric relaxation model calculations (see formula (15)).

Figure 2a shows the dependence of the real part of the dielectric constant at 1 kHz (*ε*′_1k_) on the composition ratio of the Fe+Co content in FeCo-MgF films. *ε*′_1k_ of the Mg-F film produced in this experiment is about 5.6. This value is in agreement with the value of reference bulk MgF_2_ (ref. 20). On the other hand, the value of *ε*′_1k_ of the films increases with the Fe+Co content, and attains 490 at 30 at.%. The frequency dependence of the real part of the dielectric constant (*ε*′) of the FeCo films at 14, 17, 21 and 29 at.% is presented in Fig. 2b. The *ε*′ increases with increasing Fe+Co over the whole frequency range, and exhibits a sharp decrease with increasing frequency. As discussed later, this dielectric dispersion may be caused by dielectric relaxation characterized by the relaxation time *τ*_r_. The dielectric relaxation frequency *f*_r_=1/*τ*_r_, at which *ε*′ sharply decreases, is shifted to the higher frequency side with increasing Fe+Co. As seen in Fig. 2, [Fig f3], FeCo-MgF nanogranular films have a giant dielectric constant of around 490, and *f*_r_ shifts to the higher frequency side with increasing Fe+Co. Films having giant dielectric constant and high *f*_r_ have high potentials for applications.

**Figure 3 f3:**
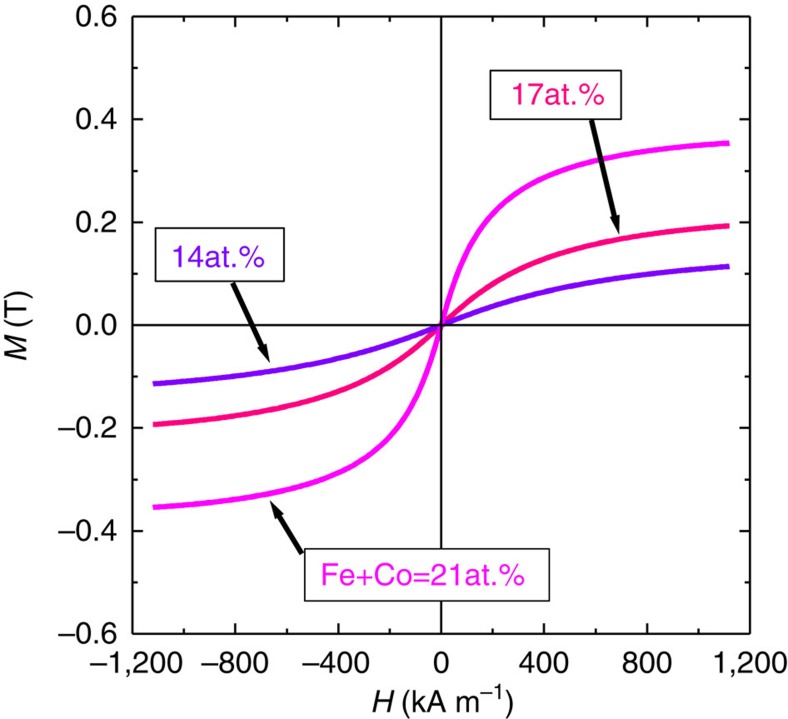
Magnetic property of FeCo-MgF nanogranular films. Magnetization curves of FeCo-MgF films for Fe+Co=14, 17 and 21 at.% measured up to an applied magnetic field of 1,200 kA m^−1^.

Figure 3 shows the magnetization curves of the Fe+Co films at 14, 17 and 21 at.%. It is understood that the films are in the superparamagnetic state, that is, their magnetization (*M*) versus magnetic field (*H*) curves follow the Langevin function and the coercivity is nearly zero^21^,^22^,^23^. This behaviour indicates that the nanogranules are uniformly dispersed. They possess magnetization at 0.1 T above room temperature.

**Figure 4:**
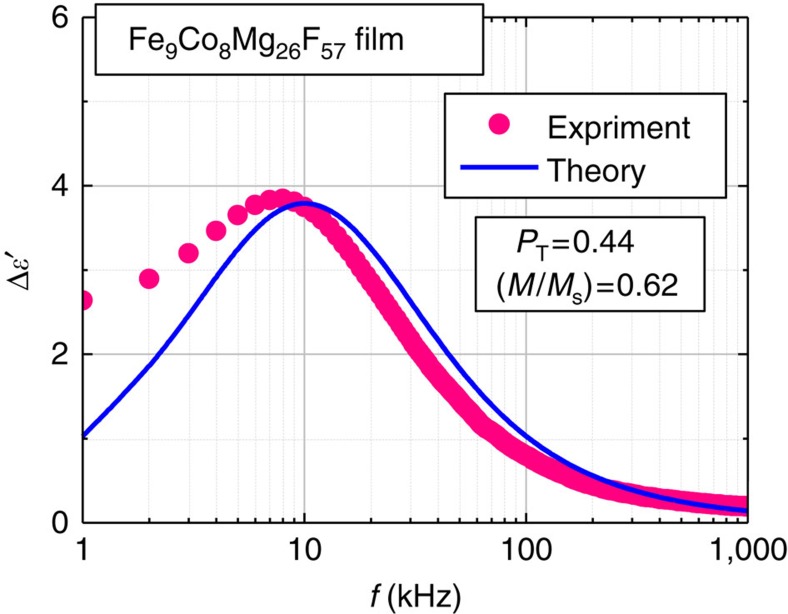
Magnetoelectric response of Fe_9_Co_8_Mg_26_F_57_ nanogranular film. Change in the dielectric constant Δ*ε*′ due to the application of magnetic field *H*=800 kA m^−1^ versus frequency *f* of the applied electric field for the Fe_9_Co_8_Mg_26_F_57_ (Fe+Co=17 at.%) film. The dielectric change Δ*ε*′=*ε*′_H_−*ε*′_0_ is the difference between the dielectric constants at magnetic field *H* and zero field *H*=0. The red dots represent the experimental results, and the blue solid lines represent the theoretical result obtained from calculations based on the spin-dependent dielectric-relaxation model (see equations (2) and (15)), where the tunnelling spin polarization^31^ *P*_T_=0.44 and the magnetization normalized by the saturation magnetization *M/M*_s_=0.62 are used.

**Figure 5:**
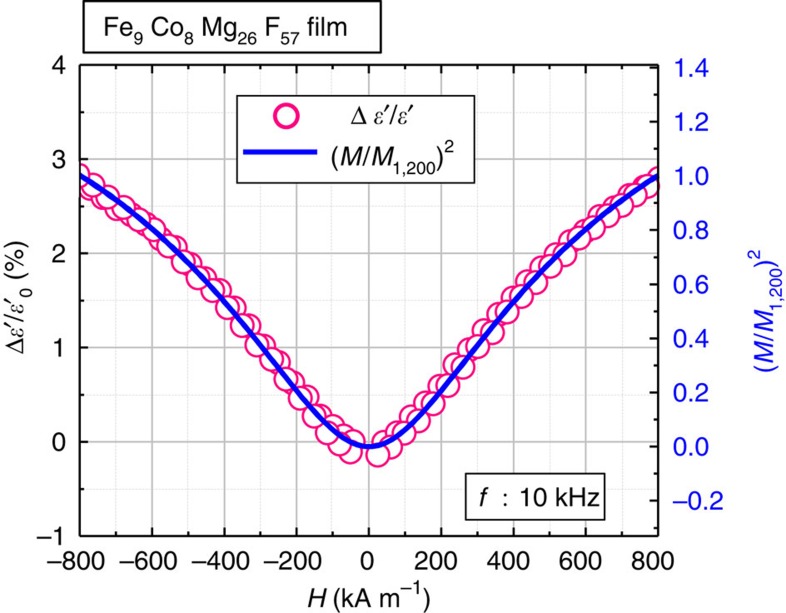
Magnetization dependence of the magnetoelectric effect of Fe_9_Co_8_Mg_26_F_57_ nanogranular film. Magnetodielectric ratio Δ*ε*′/*ε*′_0_ versus applied magnetic field *H* in the Fe_9_Co_8_Mg_26_F_57_ (Fe+Co=17at.%) film. The solid blue curve represents the values of (*M/M*_1,200_)^2^ as a function of magnetic field *H*, where the magnetization curve in Fig. 3 is used and *M*_1,200_ is the magnetization at the maximum measured magnetic field of 1,200 kA m^−1^. The DC resistivity of the film is beyond 10^10^ μΩm.

Figure 4 represents the change in the dielectric constant (Δ*ε*′) of Fe_9_Co_8_Mg_26_F_57_ (Fe+Co=17 at.%) film at applied magnetic field 800 kA m^−1^. The dielectric constant increases by the application of magnetic field over the whole frequency range, indicating a positive magnetoelectric effect. The ratio of Δ*ε*′ and the dielectric constant in zero magnetic field *ε*′_0_ (Δ*ε*′/*ε*′_0_) is plotted as a function of *H* for Fe_9_Co_8_Mg_26_F_57_ film at 10 kHz in Fig. 5. This electromagnetic effect has been observed at room temperature.

### Mechanism of dielectric and magnetoelectric responses

Dielectric and magnetoelectric responses in insulating nanogranular films may be explained by transition of the thermally activated electric charges between neighbouring granules through an insulating barrier via quantum-mechanical tunnelling^24^,^25^. The transition rate depends on the charging energy, the separation between granules, the tunnel barrier height and the relative orientation of magnetization of the granules. When thermally activated charge carriers (electrons, holes) in a granule are subject to an ac applied electric field, the electric potentials of neighbouring granules become time-varying as schematically shown in the left side of Fig. 1a. This potential oscillation gives rise to tunnelling of charge carrier back and forth through the intervening thin insulator barrier. Therefore, the oscillation of charging states between granules in the ac electric field induces electric polarization. This is the origin of the dielectric response of nanogranular films.

By incorporating the spin-dependent tunnelling process^24^,^25^ between a pair of granules into the Debye–Fröhlich model^26^ and taking into account a broad distribution of dielectric relaxation around the characteristic relaxation given by the spin-dependent tunnelling rate (1/2*τ*_r_), which is proportional to 1+*P*_T_^2^(*M*/*M*_s_)^2^ (refs 22, 25), where *P*_T_ is the tunnelling spin polarization and *M*_s_ is saturation magnetization, we obtain the complex dielectric constant of granular films


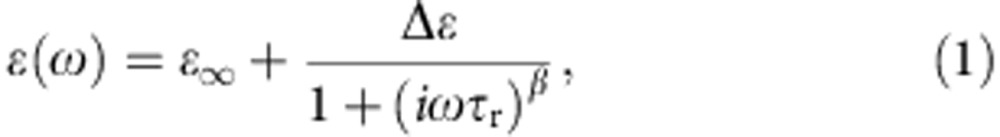


where Δ*ε* is the dielectric strength, *ε*_*∞*_ is the high-frequency dielectric constant and *β* is an exponent (0<*β*≤1) representing a measure of the distribution of relaxation time^27^. The dielectric constant *ε*′ is the real part of equation (1), and is analysed in Fig. 2b by setting *β*=0.75 and *ε*_*∞*_=10 and adjusting parameters Δ*ε* and *τ*_r_. The fitting values are Δ*ε*=185 and *τ*_r_=1.2** × **10^−4^ s, Δ*ε*=195 and *τ*_r_=1.65** × **10^−5^ s, Δ*ε*=270 and *τ*_r_=5.5** × **10^−7^ s and Δ*ε*=430 and *τ*_r_=2.2** × **10^−8^ s for Fe+Co of 14, 17, 21 and 29 at.%, respectively. We find excellent agreement between the experimental and theoretical data over the entire frequency range as seen in Fig. 2b. The strong decrease in *τ*_r_ with increasing Fe+Co content is consistent with the reduction of tunnel barrier thickness, which enhances the tunnelling rate 1/(2*τ*_r_) and explains the strong dependence of the dielectric response on the Fe+Co content shown in Fig. 2a. In addition, Δ*ε* increases with the Fe+Co content because of the increase in the number density of granule pairs (see equation (11) in Method section for details). Furthermore, using 1/*τ*_r_=(1/*τ*_r0_)[1+*P*_T_^2^(*M*/*M*_s_)^2^], we can reproduce both frequency and magnetic-field dependences of the magnetoelectric effect Δ*ε*′/*ε*′_0_ as shown in Figs 4 and 5. These results further support our model for the magnetoelectric effect in nanogranular films.

These insulating nanogranular FeCo-MgF films are a new class of multifunctional materials. These films have giant dielectric and magnetoelectric responses at room temperature with dielectric constant *ε*′=490 and magnetoelectric response Δ*ε*′/*ε*′_0_=3%. Our results suggest that the granular films can be used at higher frequencies. Our preliminary experimental results show that our films work in the digital broadcast frequency range (0.3–3 GHz) as well. Although tunable devices combining two or more materials with different properties have been proposed, these materials may be replaced with these newly discovered insulating nanogranular materials, thus producing more compact and functional devices.

## Methods

### Preparation of the thin film samples

The thin films investigated were prepared by a tandem deposition method^28^ using a conventional rf-sputtering apparatus. The sputter deposition was performed on a 50 × 50 mm^2^ glass (Corning eagle 2000) substrate in an argon atmosphere with 1.3 Pa pressure during deposition, using a Fe_60_Co_40_ alloy disk target with 76 mm diameter and a MgF_2_ powder target compacted in the form of a disc with 76 mm in diameter. To obtain a uniformly composed granular state, the substrate holder was rotated so that it alternately faced the Fe_60_Co_40_ alloy target and the MgF_2_ target. The speed of rotation was kept constant at 11.54 r.p.m. for each preparation.

### Composition and structural analysis

The composition ratio of Fe+Co (granule) and Mg+F (matrix) was controlled by changing the radio frequency (RF) power applied to each target. The chemical composition of Fe, Co, Mg and F in the thin films was analysed by wavelength dispersion spectroscopy. For the structural analysis, transmission electron microscopy was performed on selected several thin films.

### Measurements of the dielectric and magnetoelectric responses

The dielectric properties were measured by inductance, capacitance and resistance (LCR) meter with measurement range 1 kHz–1 MHz and 0–800 kA m^−1^ in magnetic field. The magnetization curves were measured by an alternating gradient magnetometer. All the measurements reported in this paper were carried out at room temperature.

### Derivation of the dielectric constant

**Figure 6:**
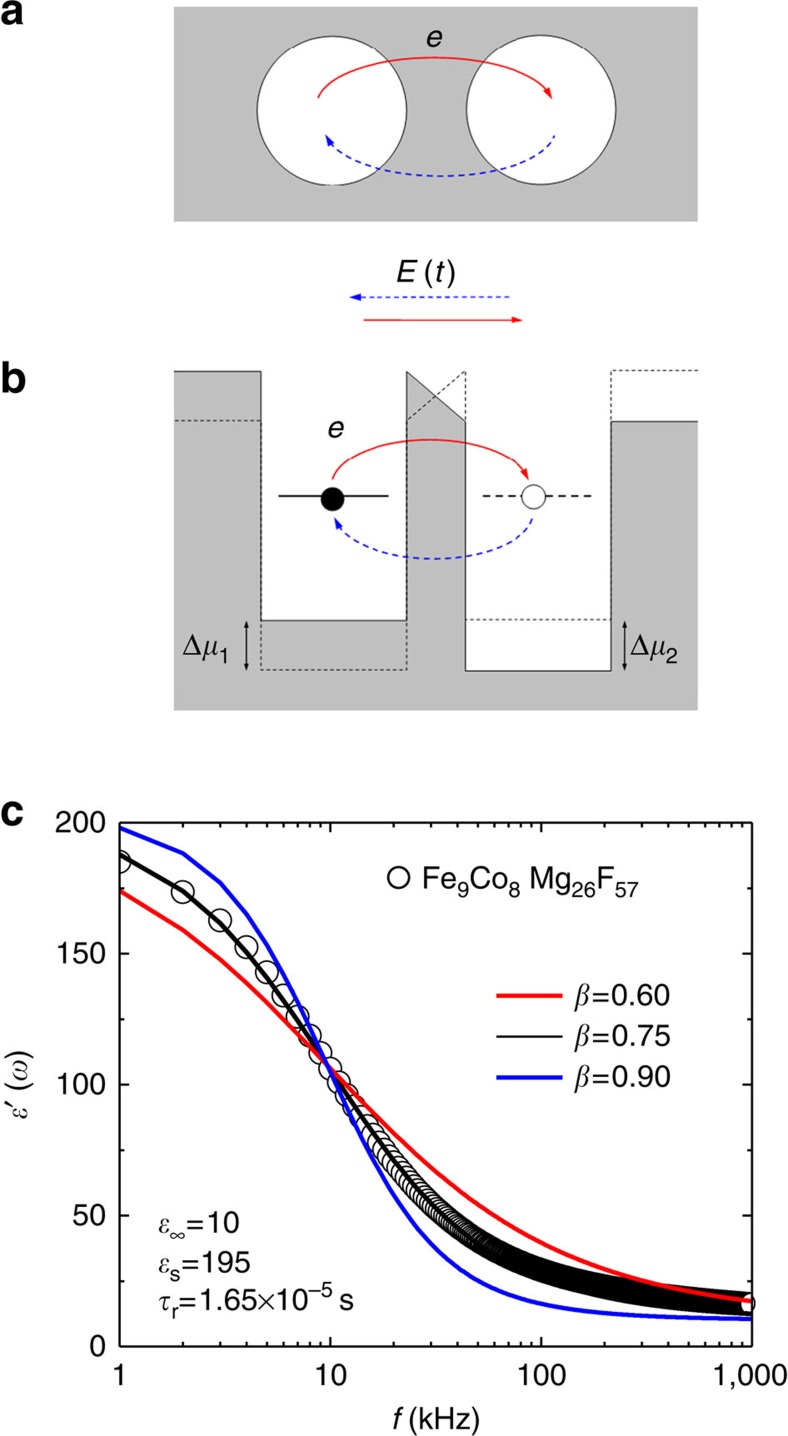
Derivation of the dielectric constant. (**a**) Ferromagnetic metal granules 1 and 2 embedded in insulating matrix. (**b**) A double potential wells illustrating an electric polarization due to tunneling of charge (electron) through the barrier from one well to the other back and forth under the ac electric field *E*(t). (**c**) The real parts of the dielectric constant are calculated from the equation (1) for three different values of *β*. The lines show the results of fitting values of *β*=0.6, 0.75, and 0.9, (Δ*_ε_*=185 (*ε_s_*=195, *ε*_∞_=10), *τ_r_*=1.65 × 10^−5^ s), and the circles represent the experimental results.

We derive the dielectric constant of a granular system composed of ferromagnetic granules of nanometer size in an insulating matrix. In a granular system, the transport is governed by thermally activated charge carriers that move from granule to neighbouring granule by tunnelling through an insulating barrier. Depending on the separation and tunnel barrier height, a charge carrier activated in one granule may tunnel to another. A simple model for a pair of granules 1 and 2 and the double potential well is schematically shown in Fig. 6a,b. In the absence of applied ac electric field, the transition rate between the two granules is determined by the charging energies and the tunnelling process^25^,^29^:


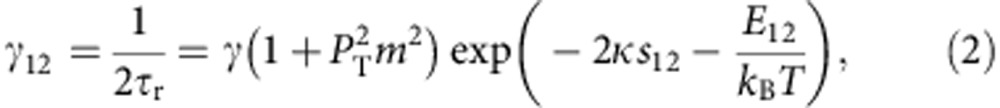


where *s*_12_ is the distance between the granular surfaces along the line connecting their centres, *m*=*M*/*M*_s_ is the magnetization of the granular system normalized to the saturation magnetization, *κ* is the decay rate of the electron wave function in the insulating barrier region of the matrix, *k*_B_ is the Boltzmann constant, *T* is the temperature and *E*_12_ is the thermal activation energy involved in the tunnelling process^29^:


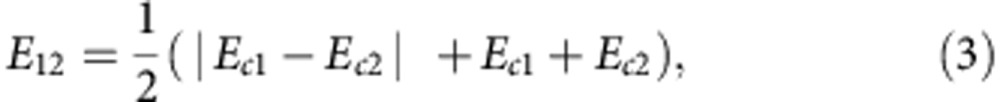


where *E*_*ci*_~*e*^2^/(*ε*_0_*d*_*i*_) (*i*=1, 2) is the charging energy of granule *i*, *ε*_0_ is the dielectric constant and *d*_*i*_ is the diameter of granule *i*.

When an ac electric field *E*(*t*) is applied, the chemical-potential shifts Δ*μ*_1_(*t*) and Δ*μ*_2_(*t*) of granule 1 and 2 become time-varying according to


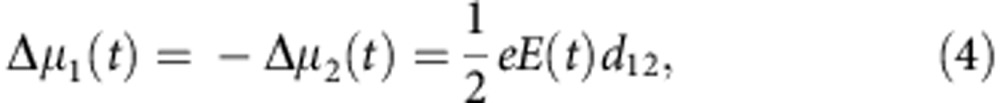


where *d*_12_ is the distance between the centres of the two granules: *d*_12_=(*d*_1_/2)+(*d*_2_/2)+*s*_12_. The time-varying electrochemical potentials give rise to the oscillation of the charging state back and forth between the granules via tunnelling of an electron. Based on the Debye–Fröhlich model^26^,^30^, the rate equations for the occupation probabilities, *P*_1_(*t*) and *P*_2_(*t*), of the thermally activated electron in granules 1 and 2 are given by


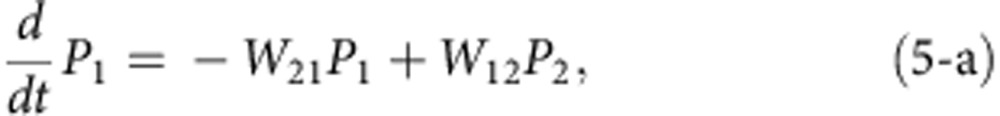



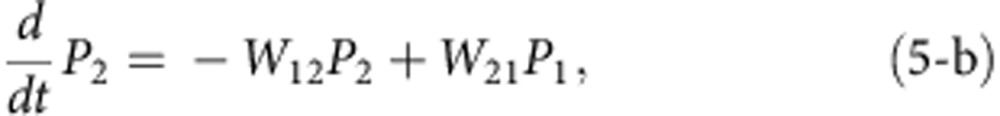


where *W*_*ij*_ are the transition probabilities:


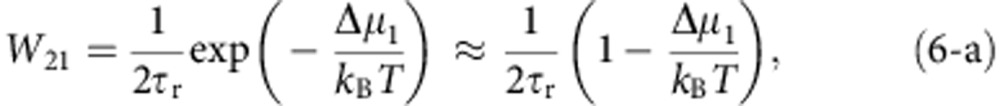



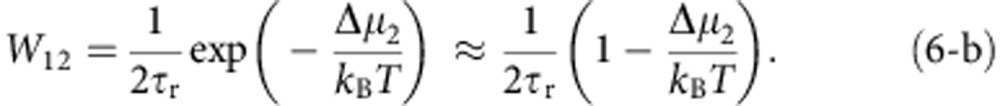


Taking the difference of (5-a) and (5-b),


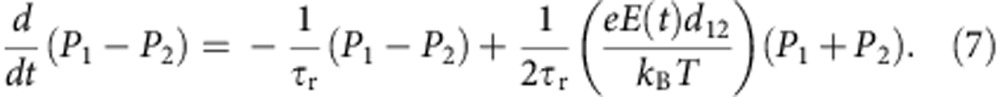


It is highly probable that a charging state is activated in either granule 1 or granule 2, for which *P*_1_+*P*_2_ is independent of time and may be given by


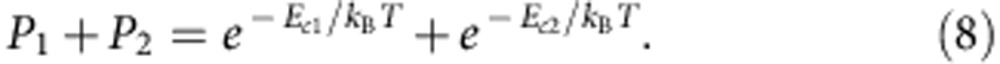


For the applied electric field *E*(*t*)=*E*(*ω*)*e*^*iωt*^, the electric polarization of the two granules is given by


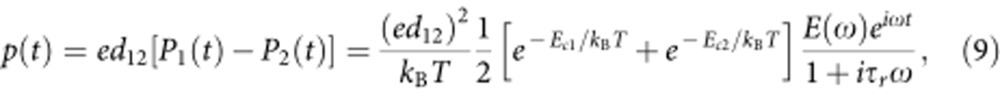


from which we can calculate the electric polarization *α*(*ω*)=*p*(*ω*)/*E*(*ω*) and therefore the dielectric constant *ε*(*ω*)=*ε*_∞_+4π*α*(*ω*) is obtained as


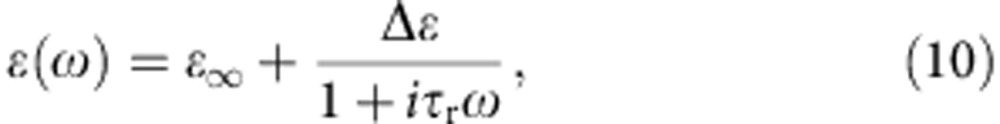


with


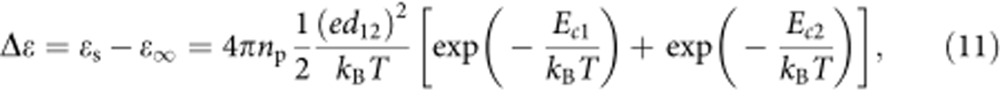


where Δ*ε*=*ε*_*s*_−*ε*_∞_ is the dielectric strength, *ε*_*s*_=*ε*(0) and *ε*_∞_=*ε*(∞) are the static and high-frequency dielectric constants, respectively, and *n*_p_ is the number density of granule pairs, which is roughly equal to the granules’ density.

The Debye–Fröhlich equation (10) above is derived from a particular pair of granules with a single relaxation time *τ*_r_. In the granular system, there are so many pairs of granules with different size and separation that the relaxation time for different granular pairs is distributed over a wide range, in which case the electric polarization may be written as


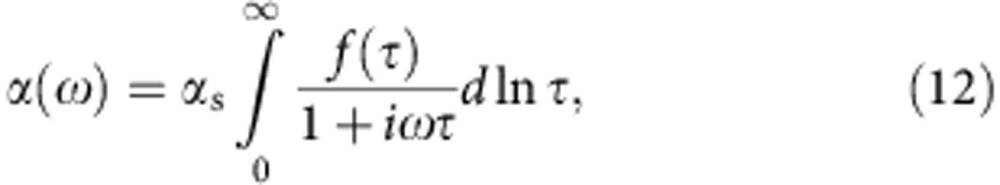


where *f*(*τ*) is the relaxation time distribution satisfying 
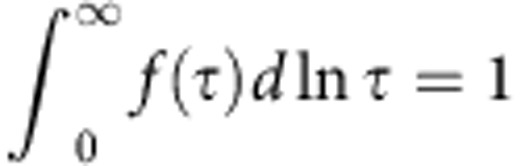
. In the case of the Debye–Fröhlich equation, the distribution function is taken as *f*(*τ*)=*δ*((*τ*/*τ*_r_)−1).

An empirical model for dielectric relaxations that takes into account the effect of a distribution of relaxation times is the equation (1) (ref. 27), where *τ*_r_ depends on the magnetization as


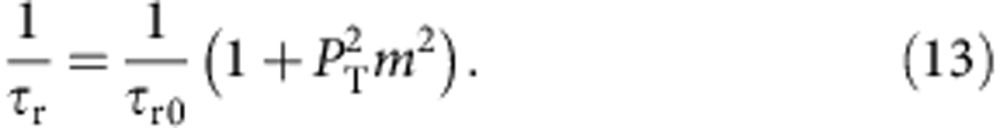


The distribution function of the Cole–Cole equation is given by^27^


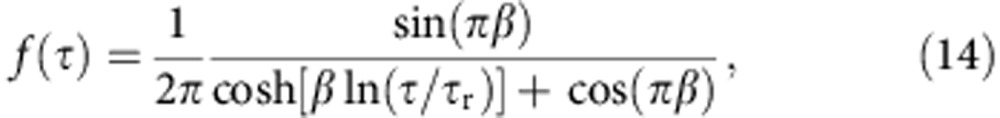


which shows that the distribution becomes broader with decreasing *β* value.

The real part *ε*′(*ω*) of the dielectric constant in equation (1) is


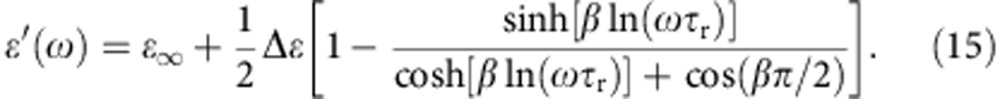


Figure 6c shows the real part of the dielectric constant as a function of frequency of applied ac electric field for *β*=0.6, 0.75 and 0.9, Δ*ε*=185 (*ε*_s_=195, *ε*_∞_=10) and *τ*_r_=1.65 × 10^−5^ s. It is clearly seen that *β*=0.75 provides an excellent fit to the experimental results of Fe_9_Co_8_Mg_26_F_57_ granular film.

## Author contributions

The experiments were carried out by N.K. The data were discussed by N.K. and H.M. The theoretical model was developed by S.T. and S.M. All authors contributed to the writing and editing of the paper.

## Additional information

**How to cite this article:** Kobayashi, N. *et al.* Giant dielectric and magnetoelectric responses in insulating nanogranular films at room temperature. *Nat. Commun.* 5:4417 doi: 10.1038/ncomms5417 (2014).
